# Hexavalent chromium (Contaminants)

**DOI:** 10.14252/foodsafetyfscj.D-1900002

**Published:** 2019-06-28

**Authors:** Food Safety Commission of Japan

## Abstract

The Food Safety Commission of Japan (FSCJ) conducted a risk assessment of hexavalent chromium, hereinafter referred to as Cr (VI), related to the amendment of the standards for beverages established by the Ministry of Health, Labour and Welfare. Major toxicities induced by Cr (VI) were damages to small intestine and anemia in experimental animals. The finding observed at the lowest LOAEL was diffuse hyperplasia of mucosal epithelium in the duodenum in mice. Regarding to carcinogenicity, Cr (VI)-treatment by drinking water significantly increased incidences of tumors in the small intestine in mice and in the oral mucosa and tongue in rats. Therefore, FSCJ considered that Cr (VI) is carcinogenic. Cr (VI) showed positive results in many genotoxic studies *in vitro*, and *in vivo* after parenteral administration, whereas no clear positive results were obtained after the oral administration. These data indicate the genotoxic properties of Cr (VI), though genotoxicity by the oral administration including drinking water remains unclear. The mechanism of small intestinal tumors in mice is considered as follows: Continuous damage to mucosal epithelium in the small intestine by long-term exposure to Cr (VI) induces the hyperplasia in the crypt of small intestine, which would lead to the formation of tumor. In the *in vivo* gene mutation assays using transgenic rats and mice, no significant increases in mutant frequencies of the transgenes were observed in the carcinogenic target tissues, after exposure to Cr (VI) in drinking water for either 28 (rats) or 90 days (mice)^1),^^2)^. On the basis of these results, FSCJ judged that the carcinogenic mechanism of Cr (VI) intakes through drinking water was hardly attributable to the genotoxicity. FSCJ considered that the quantitative risk assessment of Cr (VI) through drinking water was difficult to conduct based on the results from epidemiological studies of non-occupational and occupational exposures in human population. Consequently, specifying a tolerable daily intake (TDI), based on the results of animal studies with oral exposure to Cr (VI) through drinking water, is rather feasible. FSCJ specified the TDI of Cr (VI) as 1.1 μg/kg bw/day after applying the uncertainty factor of 100 to BMDL_10_ of 0.11 mg/kg bw/day, which was ascribed on the diffuse epithelial hyperplasia in the duodenum in male mice observed in the two-year oral exposure study. Since chromium in food is regarded to be present as trivalent chromium^3)^, FSCJ estimated daily intake of Cr (VI) from consumption of mineral water and tap water. The estimation gave the mean and high intakes as ca. 0.04 μg/kg bw/day and 0.290 μg/kg bw/day, respectively. Since both of these two values were lower than the TDI, 1.1 μg/kg bw/day, FSCJ concluded the risk of health effects from Cr (VI) at the current exposure through the consumption of mineral water and tap water to be extremely low.

## Conclusion in Brief

The Food Safety Commission of Japan (FSCJ) conducted a risk assessment of hexavalent chromium, hereinafter referred to as Cr (VI), related to the amendment of the standards for beverages established by the Ministry of Health, Labour and Welfare.

The data used in the assessment include ADME (pharmacokinetics), acute toxicity, subacute toxicity, chronic toxicity, carcinogenicity, reproductive and developmental toxicity, genotoxicity. Studies of epidemiology, mechanism for carcinogenicity in rodents, and the exposure through food and drinking water were also used. Those data were obtained from scientific articles and evaluation reports from international organizations.

Cr (VI) is absorbed at low extents after oral administration. Orally ingested Cr (VI) is reduced to trivalent chromium, slightly by saliva and mainly by gastric juice. Absorption rates of trivalent chromium are lower than that of Cr (VI). Consequently, absorption of Cr (VI) through the digestive tracts is limited. The absorbed Cr (VI) is distributed throughout tissues, then excreted mainly in urine.

Major toxicities induced by Cr (VI) were damages to small intestine and anemia in experimental animals. The finding observed at the lowest LOAEL was diffuse hyperplasia of mucosal epithelium in the duodenum in mice. Regarding to carcinogenicity, Cr (VI)-treatment by drinking water significantly increased incidences of tumors in the small intestine in mice and in the oral mucosa and tongue in rats. Therefore, FSCJ considered that Cr (VI) is carcinogenic. Cr (VI) showed positive results in many genotoxic studies *in vitro*, and *in vivo* after parenteral administration, whereas no clear positive results were obtained after the oral administration. These data indicate the genotoxic properties of Cr (VI), though genotoxicity by the oral administration including drinking water remains unclear.

The mechanism of small intestinal tumors in mice is considered as follows: Continuous damage to mucosal epithelium in the small intestine by long-term exposure to Cr (VI) induces the hyperplasia in the crypt of small intestine, which would lead to the formation of tumor. In the *in vivo* gene mutation assays using transgenic rats and mice, no significant increases in mutant frequencies of the transgenes were observed in the carcinogenic target tissues, after exposure to Cr (VI) in drinking water for either 28 (rats) or 90 days (mice)^1^^, ^^2^^)^. On the basis of these results, FSCJ judged that the carcinogenic mechanism of Cr (VI) intakes through drinking water was hardly attributable to the genotoxicity.

Accordingly, FSCJ considered that the quantitative risk assessment of Cr (VI) through drinking water was difficult to conduct based on the results from epidemiological studies of non-occupational and occupational exposures in human population. Consequently, specifying a tolerable daily intake (TDI), based on the results of animal studies with oral exposure to Cr (VI) through drinking water, is rather feasible.

Benchmark dose (BMD) approach was applied for the two-year oral exposure study through drinking water in mice. As a result, the lowest values of BMD_10_ and BMDL_10_ were derived from diffuse epithelial hyperplasia in the duodenum in male mice. The diffuse epithelial hyperplasia in the small intestine in mice above was a pre-cancerous lesion, as suggested by the mode of action (MoA) analysis of tumor development by oral exposure to Cr (VI). Therefore, FSCJ chose the pre-cancerous lesion as the critical endpoint to specify TDI.

Consequently, FSCJ specified the TDI of Cr (VI) as 1.1 µg/kg bw/day after applying the uncertainty factor of 100 to BMDL_10_ of 0.11 mg/kg bw/day, which was ascribed on the diffuse epithelial hyperplasia in the duodenum in male mice observed in the two-year oral exposure study as described above.

Since chromium in food is regarded to be present as trivalent chromium^3^^)^, FSCJ estimated daily intake of Cr (VI) from consumption of mineral water and tap water. The estimation gave the mean and high intakes as ca. 0.04 µg /kg bw/day and 0.290 µg /kg bw/day, respectively. Since both of these two values were lower than the TDI, 1.1 µg /kg bw/day, FSCJ concluded the risk of health effects from Cr (VI) at the current exposure through the consumption of mineral water and tap water to be extremely low.
